# In Silico Prediction of New Inhibitors for Kirsten Rat Sarcoma G12D Cancer Drug Target Using Machine Learning-Based Virtual Screening, Molecular Docking, and Molecular Dynamic Simulation Approaches

**DOI:** 10.3390/ph17050551

**Published:** 2024-04-25

**Authors:** Amar Ajmal, Muhammad Danial, Maryam Zulfat, Muhammad Numan, Sidra Zakir, Chandni Hayat, Khulood Fahad Alabbosh, Magdi E. A. Zaki, Arif Ali, Dongqing Wei

**Affiliations:** 1 Department of Biochemistry, Abdul Wali Khan University Mardan, Mardan 23200, Pakistan; 2 Department of Chemistry, Abdul Wali Khan University Mardan, Mardan 23200, Pakistan; 3 Department of Biology, College of Science, University of Hail, Hail 2440, Saudi Arabia; 4 Department of Chemistry, College of Science, Imam Mohammad Ibn Saud Islamic University, Riyadh 11623, Saudi Arabia; 5 Department of Bioinformatics and Biological Statistics, Shanghai Jiao Tong University, Shanghai 200240, China; 6 State Key Laboratory of Microbial Metabolism, Shanghai-Islamabad-Belgrade Joint Innovation Center on Antibacterial Resistances, Joint International Research Laboratory of Metabolic & Developmental Sciences and School of Life Sciences and Biotechnology, Shanghai Jiao Tong University, Shanghai 200030, China; 7 Zhongjing Research and Industrialization Institute of Chinese Medicine, Zhongguancun Scientific Park, Meixi, Nanyang 473006, China; 8 Henan Biological Industry Group, 41 Nongye East Rd., Jinshui, Zhengzhou 450008, China; 9 Peng Cheng National Laboratory, Vanke Cloud City Phase I Building 8, Xili Street, Nashan District, Shenzhen 518055, China

**Keywords:** KRAS G12D, machine learning-based virtual screening, molecular docking, MD simulations

## Abstract

Single-point mutations in the Kirsten rat sarcoma (KRAS) viral proto-oncogene are the most common cause of human cancer. In humans, oncogenic KRAS mutations are responsible for about 30% of lung, pancreatic, and colon cancers. One of the predominant mutant KRAS G12D variants is responsible for pancreatic cancer and is an attractive drug target. At the time of writing, no *Food and Drug Administration* (FDA) approved drugs are available for the KRAS G12D mutant. So, there is a need to develop an effective drug for KRAS G12D. The process of finding new drugs is expensive and time-consuming. On the other hand, in silico drug designing methodologies are cost-effective and less time-consuming. Herein, we employed machine learning algorithms such as K-nearest neighbor (KNN), support vector machine (SVM), and random forest (RF) for the identification of new inhibitors against the KRAS G12D mutant. A total of 82 hits were predicted as active against the KRAS G12D mutant. The active hits were docked into the active site of the KRAS G12D mutant. Furthermore, to evaluate the stability of the compounds with a good docking score, the top two complexes and the standard complex (MRTX-1133) were subjected to 200 ns MD simulation. The top two hits revealed high stability as compared to the standard compound. The binding energy of the top two hits was good as compared to the standard compound. Our identified hits have the potential to inhibit the KRAS G12D mutation and can help combat cancer. To the best of our knowledge, this is the first study in which machine-learning-based virtual screening, molecular docking, and molecular dynamics simulation were carried out for the identification of new promising inhibitors for the KRAS G12D mutant.

## 1. Introduction

Cancer is one of the primary causes of mortality globally [1]. In 2023, 1,958,310 new cancer cases and 609,820 cancer deaths are projected to occur in the United States [2]. Radiation, bacteria, and viruses account for about 7% of all cancer cases [3]. Various genetic alterations, including point mutation, deletion, and amplification, can result in the production of oncogenes [4]. Mutations in genes that play an important role in cell proliferation and differentiation are the primary cause of the majority of malignancies. Mutation in the KRAS gene is also responsible for the formation of cancer [5]. KRAS is a member of the RAS superfamily of genes and is located on chromosome 12. KRAS acts as a switch to regulate many signal transduction pathways by cycling between active and inactive states (GTP and GDP-bound, respectively). The RAF–MEK–ERK pathway is one of these signal transduction cascades [6]. The three genes (HRAS, NRAS, and KRAS) encode the four RAS proteins KRAS4A, KRAS4B, HRAS, and NRAS [7]. The two isoforms KRAS4A and KRAS4B result from the alternative splicing of exon 4, and these two isoforms have a difference in the C-terminal region [8]. However, KRAS4B is the most prevalent isoform in human cells, whereas KRAS4A expression is more comparable to viral KRAS [9]. Single-point mutations in KRAS are the most common cause of human cancer. In humans, oncogenic KRAS mutations are responsible for at least 30% of lung, pancreatic, thyroid, liver, and colon cancers [10].

Codons 12, 13, and 61 are frequently the sites of cancer-promoting KRAS mutations, with G12 accounting for the majority of these mutations (89%). Among the KRAS mutants, KRAS G12D is the most prevalent (36%) followed by KRAS G12V, (23%), and KRAS G12C (14%) [11]. The G12D variant is responsible for pancreatic cancer and is a target for drug development initiatives [12]. Because KRAS lacks binding pockets, its structure has shown to be extremely resistant to small-molecule modification [13]. To date, no FDA-approved drugs have been made available for the KRAS G12D mutant. However, one of the products of Mirati MRTX1133 is in clinical trials for patients with advanced solid tumors associated with the KRAS G12D mutation [14].

New drug development is time-consuming and expensive. It may take 10–15 years and cost up to $2 billion [14]. Conversely, in silico approaches for drug design are cost-effective and fast [15]. The drug development process has been significantly influenced by computer-assisted drug discovery (CADD) tools [16]. These in silico approaches and the advancement of supercomputing capabilities have impressively improved the effectiveness of lead discovery in pharmaceutical research [17]. Artificial intelligence (AI) and machine learning techniques are frequently used for the identification of new lead compounds [18,19]. The identification and design of new lead compounds that bind to the therapeutic drug targets are greatly enhanced by artificial intelligence and ML approaches [20].

The present study aims to identify new promising inhibitors for the KRAS G12D mutant. We used different machine learning models to identify new promising hits from the ZINC database against the KRAS G12D cancer drug target. Using Lipinski’s rule of five, drug-like compounds were selected from the ZINC database. The drug-like molecules were docked against the KRAS G12D mutant. The complexes with the top docking scores were simulated for 200 ns. The newly identified hits were found to be more stable during MD simulation. The findings indicated that these new hits may be KRAS G12D protein inhibitors, which may be important for cancer treatment.

## 2. Results

### 2.1. Preparation of Dataset

From the binding databank database, a total of 2526 compounds with reported IC50 values for KRAS G12D were obtained. Those compounds for which the IC50 value was not reported were removed from the dataset. The compounds were labeled as active or inactive based on the IC50 value of the standard compound MRTX1133 (6.1 nM) [21]. The active and inactive compounds in the dataset were denoted by the labels 1 and 0, respectively. The compound with an IC50 value less than or equal to the reference was labeled as active while the compound with an IC50 value higher than the reference was labeled as inactive. In our dataset, 422 compounds were found as active while the remaining were labeled as inactive. MOE (2016) software was employed to compute 208 2D descriptors in total. To prevent overfitting and improve the model’s generalizability, the dataset underwent preprocessing to eliminate any zero and NA values. After preprocessing, there were only 172 descriptors left.

### 2.2. Optimum Features Selection

**Figure 1:**
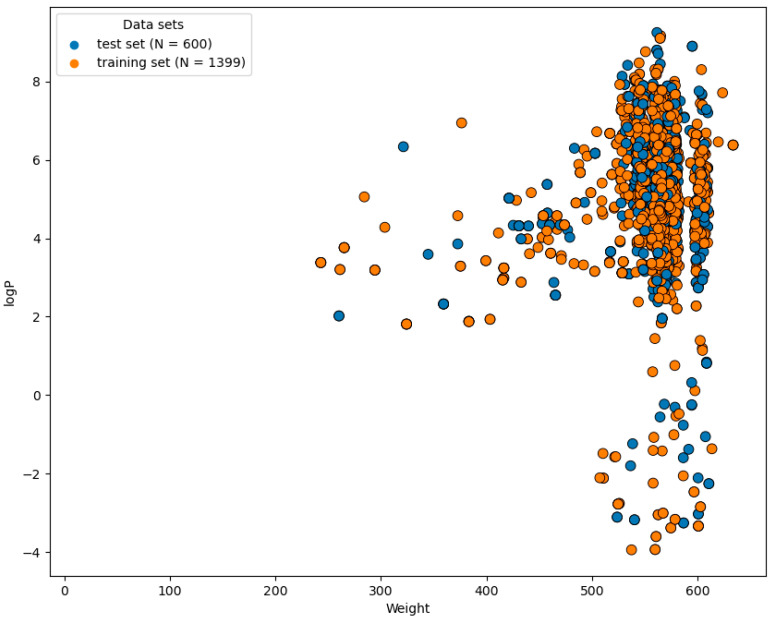
The chemical space and diversity distribution of the dataset. The scatter plot indicates the average results from the cross-validation. The molecular weight and LogP are shown on the X and Y axes, respectively.

Filter, wrapper, and embedding approaches are the three types of methods currently used by the SVM to evaluate the significance of variables in the dataset. RFE is a gold standard method among wrapper techniques [22]. In the present study, we used recursive feature elimination (RFE), for the optimum feature selection. Out of 172 features, a total of 57 optimum features including weinerPath, PEOE_VSA+2, Weight, Q_VSA_HYD, Q_VSA_POS, vdw_area, vdw_vol, vsa_hyd, SlogP_VSA0, PEOE_VSA+0, SMR_VSA6, SlogP_VSA3, Zagreb, TPSA, SMR_VSA1, SlogP_VSA7, PEOE_VSA-4, a_IC, SMR_VSA5, PEOE_VSA-0, vsa_pol, b_single, b_heavy, bpol, PEOE_VSA-1, a_heavy, SMR_VSA2, diameter, logP, weinerPol, and others were selected. Figure 1 shows the optimum feature selection curve. All machine learning models were trained using optimum feature subsets in order to increase each model’s performance.

### 2.3. Chemical Space and Diversity

The chemical diversity of a dataset significantly affects the reliability of the ML algorithm. Adequate chemical space is needed for model performance [23]. The significant chemical gap between logP and molecular weight (MW) is shown in Figure 1. A substantial chemical gap between active and inactive inhibitors was observed, with logP ranging from −4 to 8 and MW ranging from 250–600 Da, respectively.

### 2.4. Performance Evaluation of Models

**Figure 2:**
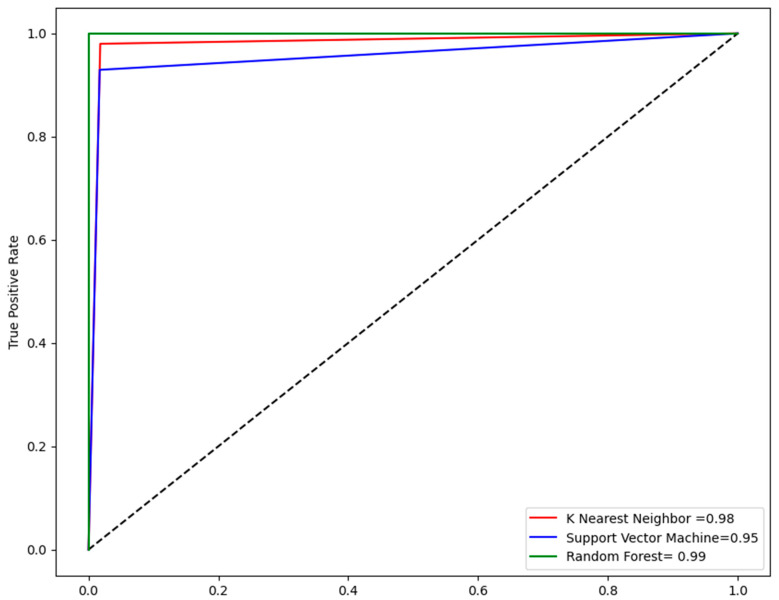
The ROC-AUC curve developed in Python v3.9 shows the TP against the FP rate on the cross-validation.

Several supervised ML models, such as KNN, SVM, and RF, were trained using Python v3.9. Several metrics like accuracy, sensitivity, specificity, and MCC were computed to access each model performance. Among all models, the accuracy of RF model was computed as 99% and the MCC value of RF model was 0.96 so it was ranked as the best model. The KNN model was ranked second based on accuracy and MCC value. The accuracy of the KNN model was found as 98% and MCC was found as 0.94. The SVM model was ranked third with an MCC value of 0.90 and an accuracy of 96%. Table 1 shows the performance evaluation of all the models. To obtain reliable results we employed five-fold cross-validation. Analyzing the ROC-AUC curve is one of the most reliable methods to assess model performance. With an area under the curve (AUC) value of 0.99 the RF model outperformed the KNN and SVM models, with an AUC value of 0.98 and 0.95, respectively, as shown in Figure 2.

### 2.5. Virtual Screening

Among the ML algorithms, the RF model revealed good accuracy and MCC score so it was used for the virtual screening of a total of 20,000 drug-like compounds retrieved from the ZINC database. A total of 82 hits were predicted as active against the KRAS G12D mutant. Among these 82 hits, ten hits were found to be toxic, so these compounds were removed from the database while the non-toxic compounds were docked against the KRAS G12D mutant.

### 2.6. Molecular Docking Study

**Figure 3:**
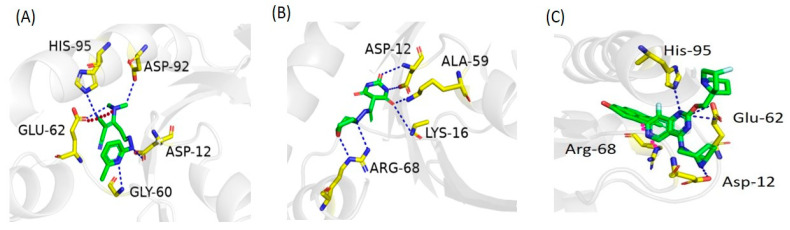
Three-dimensional interactions of (**A**) ZINC05524764, (**B**) ZINC05828661, and (**C**) the control compound with the KRAS G12D mutant. The blue dotted lines indicate hydrogen bonds, the red dotted line indicates the ionic bond, and the pink dotted line indicates the arene-cation bond, while ligands are shown as green sticks.

All 72 hits were docked into the active site of the KRAS G12D mutant. The docking analysis revealed that most of the newly identified hits revealed good docking scores and interactions with the KRAS G12D mutant. MRTX-1133 was selected as the control compound in the docking study. Compound ZINC05524764 was identified as the most promising with a docking score of −7.91 (kcal/mol). Compound ZINC05524764 establishes five hydrogen bonds with Glu62, Asp92, Asp12, His95, and Gly60 and one ionic interaction with Glu62 residues of KRAS G12D. Compound ZINC05828661 was found to be the second most potent compound with a docking score of −6.85 (kcal/mol). Compound ZINC05828661 made six hydrogen bond interactions with the Asp12, Lys16, Ala59, and Arg68 active site residues. The docking score of compound ZINC05725307 was predicted as −6.70 (kcal/mol). Compound ZINC05725307 made three hydrogen bond contacts with Asp12 and Arg102 and one ionic interaction with Lys16, one arene-H interaction with Ala59, and one arene-cation interaction with Arg68 residue of the KRAS G12D receptor. Control compound MRTX1133 revealed four hydrogen bonds with the Asp12, Glu62, and His95 active site residues of KRAS G12D while one arene-cation interaction with Arg68 was also observed. Table 2 shows the docking score and interactions of the most promising hits of the ZINC database. The 3D interactions of the most promising compounds in comparison with the control compound are shown in Figure 3.

### 2.7. Docking Validation

**Figure 4:**
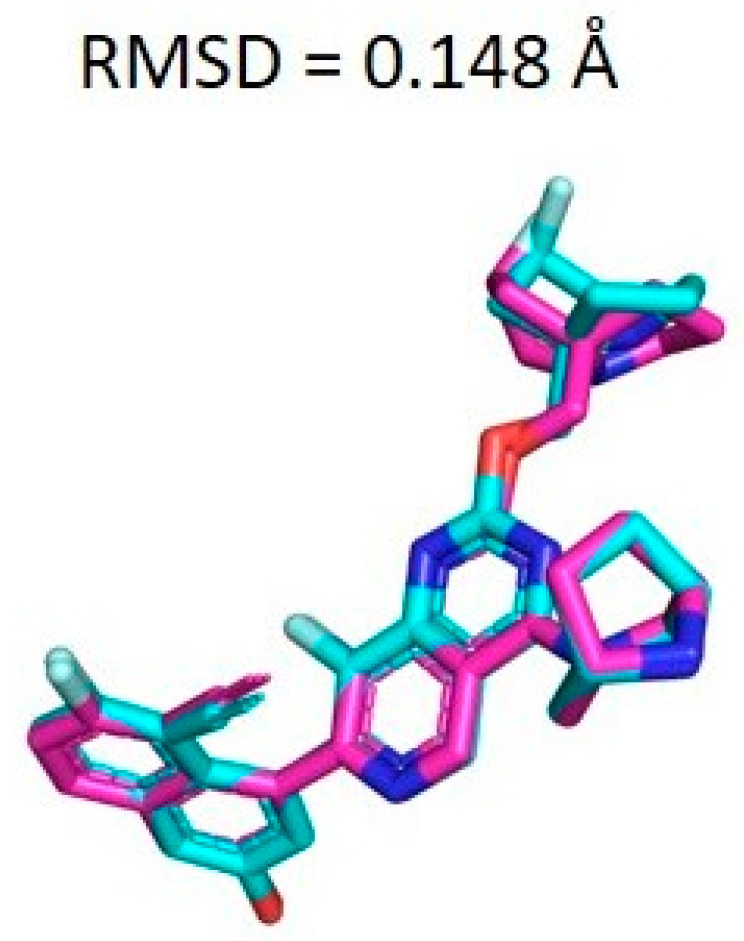
Superposition of co-crystallized and docked conformations of the ligand. The magenta color represents the native co-crystallized ligand and the cyan color is the docked ligand.

The docking procedure was validated by removing the co-crystal ligand (PDB ID: 7RPZ) and then re-docking it into the active site using MOE (2016) software [23]. The RMSD value between the top-ranked docked conformation and the co-crystallized ligand was predicted to be 0.148 Å (Figure 4), revealing the validity of the MOE docking protocol.

### 2.8. Drug-Likeness and Toxicity Analysis of the Compounds

In evaluating the drug-likeness of the compounds, one widely accepted criterion is the Lipinski rule of five. In this study, the MOE software was employed to calculate the drug-likeness of the compounds. The Lipinski rule of five for the most promising compounds is present in Table 3. All the compounds obeyed the Lipinski rule of five. Our newly identified compounds against the KRAS G12D target possess drug-likeness. Furthermore, the virtual toxicity of the compounds was evaluated by using the MOE software. All the compounds were predicted non-toxic as presented in Table 4.

### 2.9. Post-Simulation Analysis

#### 2.9.1. RMSD Analysis

**Figure 5:**
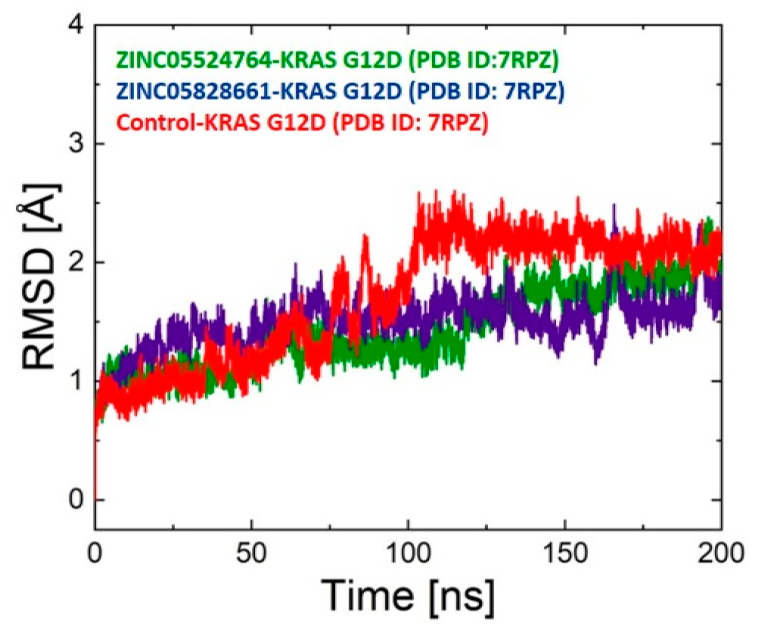
RMSD plot for ZINC05524764 (green), ZINC05828661 (purple), and the control (red) systems. Time in ns is shown on the X-axis and the RMSD value of each system is shown on the Y-axis.

One of the most acceptable methods for examining the underlying stability of protein-ligand complexes is the performance of MD simulations. The stability of the complexes was evaluated by RMSD analysis. For the 200 ns production simulations, the RMSD of the KRAS G12D was plotted and the result was compared to the control complex. The RMSD of the ZINC05524764 complex was initially stable up to 50 ns but minor fluctuations were observed between 50 and 55 ns then the system converged and remained stable to 120 ns. After 120 ns, the RMSD gradually increased up to 170 ns, then the system attained stability and remained stable up to 200 ns. The RMSD of the ZINC05828661 complex revealed stability during the first 50 ns, after that minor deviations were seen between 50 and 70 ns, then the system attained stability and remained stable up to 200 ns, except for some deviation between 125 and 175 ns. However, when compared to the control system, the RMSD of the two systems were found to be highly stable during the 200 ns MD simulation. The control system revealed unstable behavior between 60 and 125 ns but overall, a stable RMSD was observed for all the systems. The average RMSD of the ZINC05524764, ZINC05828661, and control systems was found to be 2 Å, 2.1, and 2.5 Å, respectively. Figure 5 displays the RMSD plots for all of the complex systems. The ligand RMSD also showed limited fluctuation, indicating that once bound, the ligand remains consistently positioned within the binding site of the KRAS G12D protein. The minimal deviation of the RMSD ligand from the RMSD complex suggests a synergistic stability between the ligand and the protein, an indication of a stable complex that is less likely to dissociate under physiological conditions. This result suggests that ZINC05524764 has the potential to act as an inhibitor for the KRAS G12D protein. Figure S1 shows the RMSD ligand plots, while Figure S2 shows the complex systems before and after MD simulation.

#### 2.9.2. RMSF Analysis

**Figure 6:**
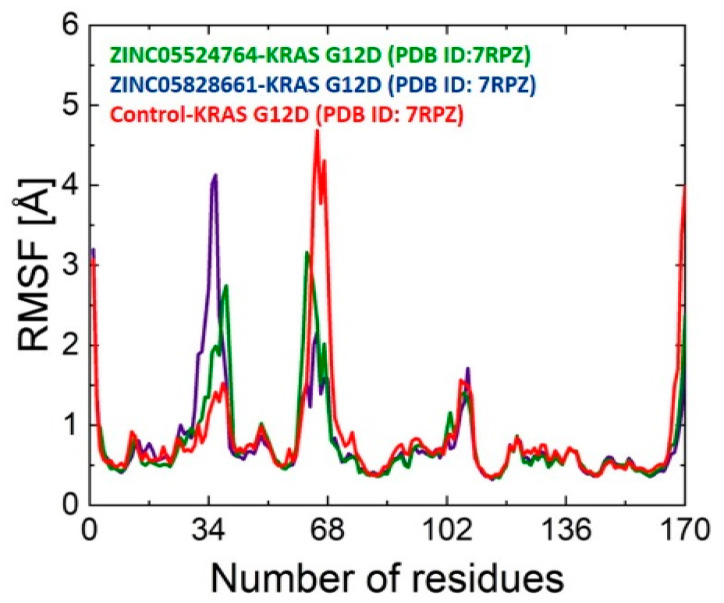
RMSF plot for ZINC05524764 (green), ZINC05828661 (purple), and the control (red) systems. The number of residues is displayed on the X-axis and the RMSF value of each system is present on the Y-axis.

The root mean square fluctuation (RMSF) allowed for a more thorough examination of the protein’s backbone flexibility. The RMSF plots for all the complexes are shown in Figure 6. The loop regions had the highest variations, with an overall comparable tendency in the fluctuations. Residues Asp30, Glu31, Tyr32, Asp33, Pro34, Thr35, Ile36, Ser65, Ala66, Met67, Arg68, and Asp69 revealed high fluctuations during MD simulation. Conversely, a decrease in flexibility was noted in the region where the inhibitor was bound, indicating the impact of inhibitor interactions with the active site residues of KRAS G12D.

### 2.10. Structure Compactness Analysis

**Figure 7:**
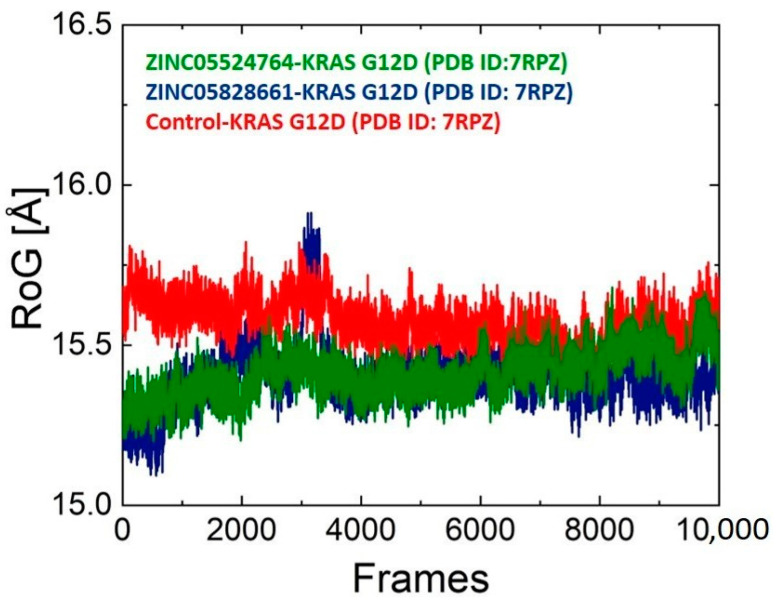
RoG plot for ZINC05524764 (green), ZINC05828661 (purple), and the control (red) systems. The number of frames and the RoG value are presented on the X and Y axis.

We calculated the structural compactness in a dynamic setting to determine the binding and unbinding processes that took place during the simulation. The radius of gyration (Rg), as a function of time, was used to evaluate the structural compactness. The Rg of ZINC05828661 showed a similar trend to that of RMSD, as shown in Figure 7. For a short period in the first 50 ns, the complex first reported low Rg values. After that, the Rg value increased to 15.9 Å, then decreased again, and continued to follow a consistent pattern up to 200 ns. The average Rg value for the ZINC05524764 system (green) was found to be 15.2–15.6 Å, the Rg value for the ZINC05828661 system was observed to be 15.1–15.8 Å, and for the control system, the Rg value was found to be 15.3–15.7 Å. Figure 6 displays the Rg plots for all the systems.

#### DCCM Analysis

**Figure 8:**
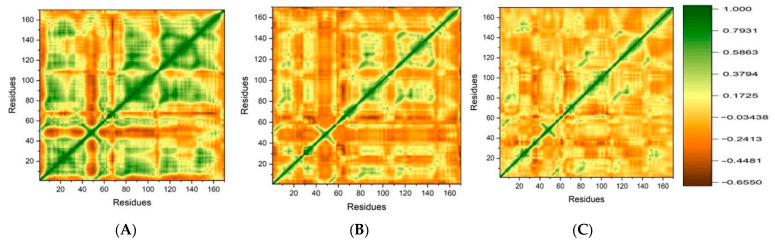
DCCM plot for the (**A**) ZINC05524764, (**B**) ZINC05828661, and (**C**) control systems. The X and Y axis shows the number of residues.

By computing the correlation among residues of receptor the dynamic cross-correlation map (DCCM) was employed to obtain information regarding correlated motions during the MD simulation. Inter-residue correlation analysis, or DCCM, was carried out to elucidate the correlations among the residues in the systems. Figure 8 displays the DCCM results for all of the complex systems. The motions of the amino acids appeared positively correlated, indicating that they were strongly associated with correlated motions. If the amino acids are moving in the opposite or reverse direction, demonstrated anti-correlations of motion. The anti-parallel and parallel directions, respectively, represent the negative and positive correlations between the residues of the systems [24]. The dark brown region in the plots shows a negative correlation while the green regions indicate positive correlations between the residues. More positive correlations were observed in ZINC05524764 and ZINC05828661, as compared to the control system.

### 2.11. Binding Energy Calculation

Using the binding free energy method, or MM-GBSA, to measure the binding strength of small molecules is a frequently used technique to confirm the ligand binding and docking stability. In terms of calculation, the MM-GBSA approach which was previously reported is less expensive and, as compared to the rational scoring functions, is one of the most accurate techniques [25]. We also used this method to determine the binding free energy for the ZINC05524764, ZINC05828661, and control complexes, keeping in mind its applicability. Total binding free energy (TBFE) estimates for the ZINC05524764 complex were −39 kcal/mole, for the ZINC05828661 complex the binding energy was calculated as −35 kcal/mole, and for the control system, the binding free energy was found as −30 kcal/mole. Table 5 shows the results of the MMGBSA analysis.

## 3. Discussion

The second most common cause of cancer death is considered to be pancreatic ductal adenocarcinoma (PDAC) in the US. For metastatic PDAC, the 5-year survival rate is less than 5% due to the restricted therapeutic choices available [26,27]. Human malignancies are often linked to the activation of missense mutations of RAS genes (KRAS, HRAS, and NRAS), which are crucial in oncogenic transformation [28]. Due to the absence of binding sites appropriate for small-molecule inhibitors, oncogenic RAS proteins have long been thought to be undruggable [29]. Most KRAS mutations occur at codon 12, where G12D mutations account for the largest frequency (35%), followed by G12V (20–30%), G12R (10–20%), Q61 (~5%), G12C (1–2%), and other uncommon mutations. [30] FDA has approved sotorasib (AMG510) and adagrasib (MRTX849) for the treatment of advanced lung cancer with a KRASG12C mutation. Additionally, MRTX 1133, a KRAS G12D inhibitor, has demonstrated encouraging preclinical development outcomes, and it is presently undergoing a phase 1 clinical trial. To date, no FDA-approved drugs are available for the KRAS G12D mutant. So, there is a need to develop a new and effective drug for KRAS G12D [31]. The pharmaceutical industry has benefited greatly from the deployment of several machine learning algorithms in drug discovery. Predicting bioactivity, drug–protein interactions, and enhancing the bioactivity and safety profile of compounds are among the common uses of these algorithms [32]. For the identification of new inhibitors against different drug targets, ML-based virtual screening is widely used [33,34].

In this study, different machine learning models were used to identify new promising hits from the ZINC database against the KRAS G12D cancer drug target. Among the 82 hits predicted as active, a total of 10 hits were found to be toxic. These toxic compounds were removed, and the remaining hits were docked into the active site of KRAS G12D. The molecular docking analysis confirmed six compounds as the most promising inhibitors for KRAS G12D. A previous study identified three promising inhibitors Quercetin, Psoralidin, and Resveratrol for the KRAS G12D mutant. These promising inhibitors formed hydrogen bonding with the Gly10, Thr58, Asp69, Tyr96, Gln61, Glu62, Tyr64, Met72, and Arg68 active site residues of KRAS G12D [35]. Our promising inhibitors also made interactions with the active site residues including Gly10, Asp12, Lys16, Thr58, Glu62, Gly60, Arg68, Met72, and His95. Following molecular docking, a 200 ns MD simulation was carried out for the top two complexes along with the standard complex to determine their stability. The identified hits revealed stable binding to the protein confirmed by the RMSD analysis, demonstrating that these compounds are appropriate inhibitors of KRAS G12D. The stability of the ZINC05524764 complex in comparison to all other complexes was further corroborated by the RoG analysis, which is consistent with the RMSD profile. Furthermore, MMGBSA analysis revealed the strong binding energy of the two complexes as compared to the control complex.

## 4. Materials and Method

### 4.1. Dataset Preparation

A total of 2526 compounds for the KRAS G12D mutant found in the Binding DB were extracted. MRT1133 was considered as the standard compound. The standard compound’s IC50 value was found to be 6.1 nM [21]. Based on the IC50 value, the compounds were divided into active and inactive categories. For 526 compounds, the IC50 value was not reported so these were removed. A total of 1578 compounds were categorized as inactive because their IC50 value exceeded that of the reference compounds, while 422 compounds were considered active because their IC50 value was equal to or less than that of the reference compound. In the target class, the active and inactive compounds were indicated by 1 and 0, respectively.

### 4.2. Features Extraction and Dataset Cleaning

The experimentally validated compounds against the KRAS G12D mutant were obtained from Binding DB. Then, descriptors were calculated in MOE (2019) software [36]. A total of 206 features were computed by MOE software. All the 0 and null (NA) values were removed from the dataset using python v3.9. The dataset cleaning was carried out using the pandas library of python [37]. Then, the dataset was split into training (70%) and test (30%) subsets. The train_test_split function was used to divide the dataset into training and test sets [38].

### 4.3. Feature Selection

To develop a computationally inexpensive model and to improve model performance, optimum features selection was carried out. We employed SVM-RFE to choose optimum features for model development [39].

### 4.4. ML Models

Using open-source Python v3.9, three models such as the k-nearest neighbors, support vector machine, and random forest models were developed. All the models were developed using the scikit-learn package of the Python software v3.9 [23].

### 4.5. K-Nearest Neighbor (kNN)

The k-nearest neighbors (KNN), also known as a lazy algorithm, can solve the problems of classification as well as regression. First, the distance between the nearest neighbors in the data can be measured [40]. The parameter n_neighbors can be used to select the nearest neighbors [41]. The optimal k value was found to be 11.

### 4.6. Support Vector Machine (SVM)

The SVM model can tackle the problems of regression and classification [42]. Apart from binary classification, SVM can address multiclass classification problems. SVM classifies data with the help of an optimum hyper-plane. Various kernel functions (linear, polynomial, sigmoid, and radial base functions) are used to convert low-dimensional data into a higher dimensional space [43]. The grid search method and RBF were employed to predict the optimal values for the C and γ parameters. Finally, C = 1000 and γ = 1 were found to be the ideal values.

### 4.7. Random Forest (RF)

The RF algorithm was first presented by Breiman [44]. It is a favored model for data categorization or regression tasks. A bootstrap sample is used to train the random forest tree, and predictions are made by the majority vote of the trees. Max_features and n_estimators, which indicate the number of trees built before predictions, were the two main hyperparameters that were optimized during model development [41]. Some 100 to 500 estimates were taken during model generation.

### 4.8. Models Validation and Performance Evaluation

In the case of unbalanced datasets, accuracy alone is not sufficient to access the strength of a classification model [45]. In the case of binary classification problems, the MCC parameter can be used to evaluate the performance of a model. The receiver operating characteristic (ROC) curve is another useful tool for evaluating the models’ performance. A ROC curve can be used to visually represent the true positive rate against the false positive rate [46]. For ML model evaluation, several parameters were calculated, including accuracy, F1 score, MCC score, and ROC curves. We employed five-fold cross-validation in this study.

### 4.9. Virtual Screening and Molecular Docking Study

The model that revealed high accuracy and MCC values was used for the virtual screening of the 20,000 drug-like compounds of the ZINC database [47]. The hits obtained from the RF model were docked against the KRAS G12D mutant. The 3D structure of the KRAS G12D mutant (PDB ID: 7RPZ) was retrieved from the PDB database. The water molecules were removed from the structure before docking [48]. Energy minimization was carried out using an RMS gradient of 0.05. The protein preparation module of the MOE version 2016 (Chemical Computing Group, Montreal, QC, Canada) software was used to prepare the structure. The KRAS structure was 3D protonated. Ten conformations were generated in total for each hit [49]. Finally, for docking analysis, the PyMOL version 2.5 (Schrödinger, New York, NY, USA) and MOE version 2016 (Chemical Computing Group, Montreal, QC, Canada) software were used.

### 4.10. MD Simulation

Using the AMBER version 2022 (Schrödinger, San Francisco, CA, USA) package [24], MD simulation was carried out for 200 ns to examine the stability and dynamic evaluation of the best complexes. For protein and ligand molecules, the FF19SB force field and the general amber force field (GAFF), respectively, were used [50]. Na^+^ ions were added to counteract the effects of any charge, and energy reduction was accomplished in two phases (using the steepest descent and conjugate gradient methods) [51]. The heating and equilibration processes were then carried out. Then, the production run of 200 ns for each complex was run. The particle mesh Ewald algorithm was applied to the long-range electrostatic interactions using cutoff distance of 10.0 Å [52]. Lastly, the simulations were conducted using PMEMD.cuda, and the trajectories were analyzed using the CPPTRAJ package [53].

### 4.11. Binding Free Energy Calculations

The most frequently utilized method in various research studies is the assessment of the potency of small molecule binding by calculating the binding free energy (BFE) using the MM/GBSA approach [54]. We employed the MMPBSA.py script to calculate the binding free energy of the protein–ligand complexes by taking into account 2500 snapshots. To calculate the BFE, the following formula was applied:

∆*G bind* = ∆*G complex* − [∆*G receptor* + ∆*G ligand*]

The binding energy of a protein, drug, or complex is represented by the symbols ∆*G receptor*, ∆*G ligand*, and ∆*G complex*, respectively, while the overall binding energy is represented by the symbol ∆*G bind* [25].

## 5. Conclusions

The KRAS G12D variant is responsible for pancreatic cancer and is a target for cancer drug development initiatives. In this study, different computational approaches were used to identify new promising inhibitors for the KRAS G12D mutant. Among the 72 active hits against KRAS G12D, two compounds ZINC05524764 and ZINC05828661 were found to be the most promising for the KARS G12D mutant. As compared to the standard compound MRTX 1133, our reported compounds revealed high stability during the 200 ns MD simulation. Our identified hits have the potential to inhibit the KRAS G12D mutation and can help combat cancer. This study provides hope for the development of new drugs to treat the cancer caused by the KRAS G12D mutation. This work sets the stage for continued innovation in the field of drug discovery. It is further recommended to evaluate the inhibitory potential of these compounds through in vitro and in vivo approaches.

## Figures and Tables

**Table 1:** Performance evaluation of machine-learning models.

Models	Accuracy	Sensitivity	F1 Score	MCC
KNN	98	0.99	0.95	0.94
SVM	96	0.93	0.92	0.90
RF	99	0.94	0.96	0.96

**Table 2:** Docking score and interactions of the most potent compounds of ZINC database.

Zinc ID	Interacting Residues	Interaction Type	Distance (Å)	Energy (kcal/mol)	S Score (kcal/mol)
ZINC05524764	GLU 62	H-bond	3.30	−2.0	−7.91
	ASP 92	H-bond	3.13	−1.8	
	ASP 12	H-bond	3.02	−2.1	
	HIS 95	H-bond	2.96	−2.8	
	GLY 60	H-bond	3.23	−3.5	
	GLU 62	Ionic	3.72	−1.1	
ZINC05828661	ASP 12	H-bond	3.01	−2.6	−6.85
	LYS 16	H-bond	3.15	−1.7	
	Ala 59	H-bond	3.25	−0.6	
	ASP 12	H-bond	3.30	−0.5	
	ARG 68	H-bond	3.20	−2.6	
	ARG 68	H-bond	3.23	−1.5	
ZINC05725307	ASP 12	H-bond	2.88	−1.6	−6.70
	ARG 102	H-bond	2.88	−5.1	
	LYS 16	H-bond	3.33	−0.9	
	LYS 16	Ionic	2.78	−6.2	
	ALA 59	Arene-H	4.12	−0.6	
	ARG 68	Arene-cation	4.83	−0.8	
ZINC17004657	GLN 61	Arene-H	3.88	−1.1	−5.68
	ASP 12	H-bond	2.98	−1.6	
	ASP 12	H-bond	3.05	−1.2	
	LYS 16	H-bond	3.30	−1.0	
ZINC18169629	GLN 61	H-bond	3.09	−0.6	−6.19
	HIS 95	H-bond	2.91	−6.2	
	GLY 60	H-bond	3.26	−1.0	
	LYS 16	H-bond	3.13	−3.0	
	ALA 59	Arene-H	4.03	−1.2	
	GLY 60	Arene-H	4.39	−0.6	
	THR 58	Arene-H	4.02	−0.8	
ZINC22760692	GLU 63	H-bond	3.20	−1.1	−6.51
	HIS 95	H-bond	3.24	−0.8	
	ARG 68	H-bond	3.12	−0.5	
	GLY 10	H-bond	3.10	−0.5	
	LYS 16	H-bond	3.16	−0.8	
	MET 72	Arene-H	4.17	−0.6	
Control	GLU 62	H-bond	3.29	−1.4	−5.39
	GLU 62	H-bond	3.30	−0.7	
	ASP 12	H-bond	2.64	−3.1	
	HIS 95	H-bond	2.77	−3.0	
	ARG 68	Arene-cation	4.72	−0.7	

**Table 3:** Drug-likeness of the compounds.

Compound ID	M-Weight	HB-Donor	HB-Acceptor	logP
ZINC05524764	254.25	3	5	−1.41
ZINC05828661	289.75	2	4	0.13
ZINC05725307	259.24	3	4	0.41

**Table 4:** Two-dimensional structures and toxicity analysis of the most promising compounds.

Compound ID	2D Structure	Toxicity
ZINC05828661	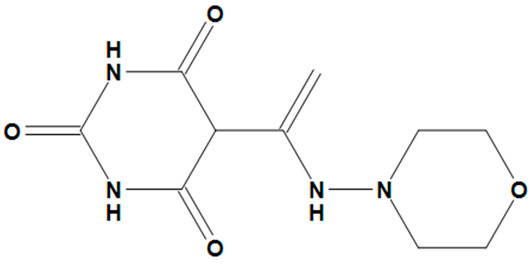	No
ZINC05524764	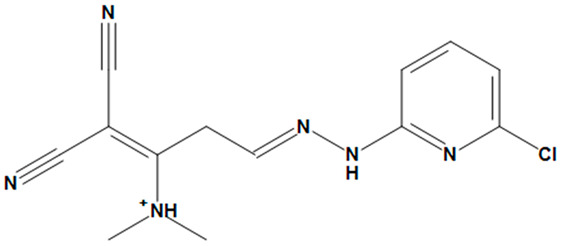	No
ZINC05725307	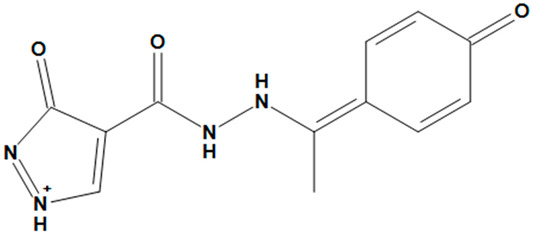	No

**Table 5:** MMGBSA analysis indicating the binding energy of all the complexes.

Complex	vdW	EEL	ESURF	EGB	Δ*G* TOTAL
ZINC05524764-KRAS^G12D^	−48.7803	−9.8255	−5.8669	25.3835	−39.0880
ZINC05828661-KRAS^G12D^	−42.7893	−5.4652	−4.8129	17.8249	−35.2418
Control-KRAS^G12D^	−26.6921	−29.9760	−4.5080	30.4723	−30.7021

## Data Availability

Data are contained within the article.

## Supplementary Materials

The following supporting information can be downloaded at: https://www.mdpi.com/article/10.3390/ph17050551/s1, Figure S1: RMSD plot for ligands ZINC05524764 (Green) ZINC05828661 (Purple) and Control (Red) systems. Time in ns is shown on the X-axis and the RMSD value of each ligand is shown on the Y-axis; Figure S2: (A–C) indicates the complex ZINC05524764, ZINC05828661, and Control systems before MD simulation while (D–F) indicates the ZINC05524764, ZINC05828661, and Control systems after MD simulation.
