# Supplementary material for: In Silico Prediction of New Inhibitors for Kirsten Rat Sarcoma G12D Cancer Drug Target Using Machine Learning-Based Virtual Screening, Molecular Docking, and Molecular Dynamic Simulation Approaches
Source: Pharmaceuticals (Basel). 2024 Apr 25;17(5):551. doi: 10.3390/ph17050551 (PMC11124053; doi:10.3390/ph17050551)
Supplement: Supplementary file 1 [file pharmaceuticals-17-00551-s001.zip › pharmaceuticals-2916784-supplementary.pdf]

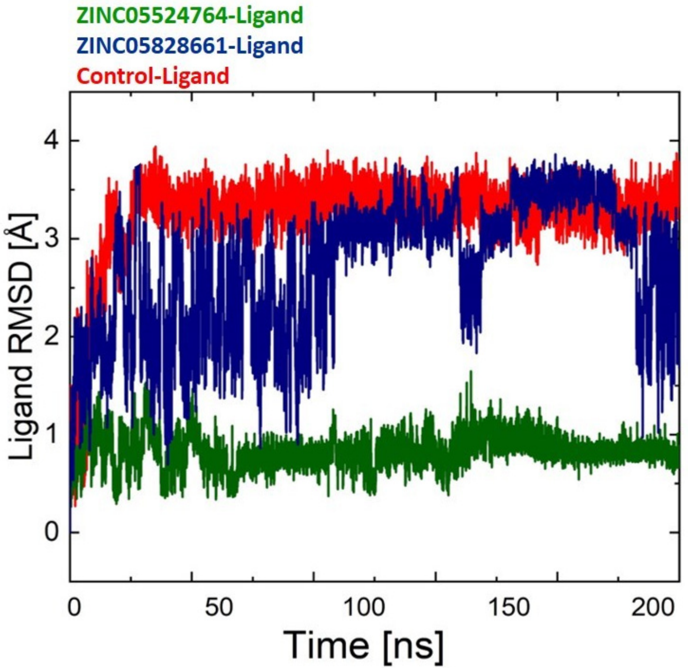

Figure S1. RMSD plot for ligands ZINC05524764 (Green) ZINC05828661 (Purple) and Control (Red) systems. Time in ns is shown on the X-axis and the RMSD value of each ligand is shown on the Y-axis.

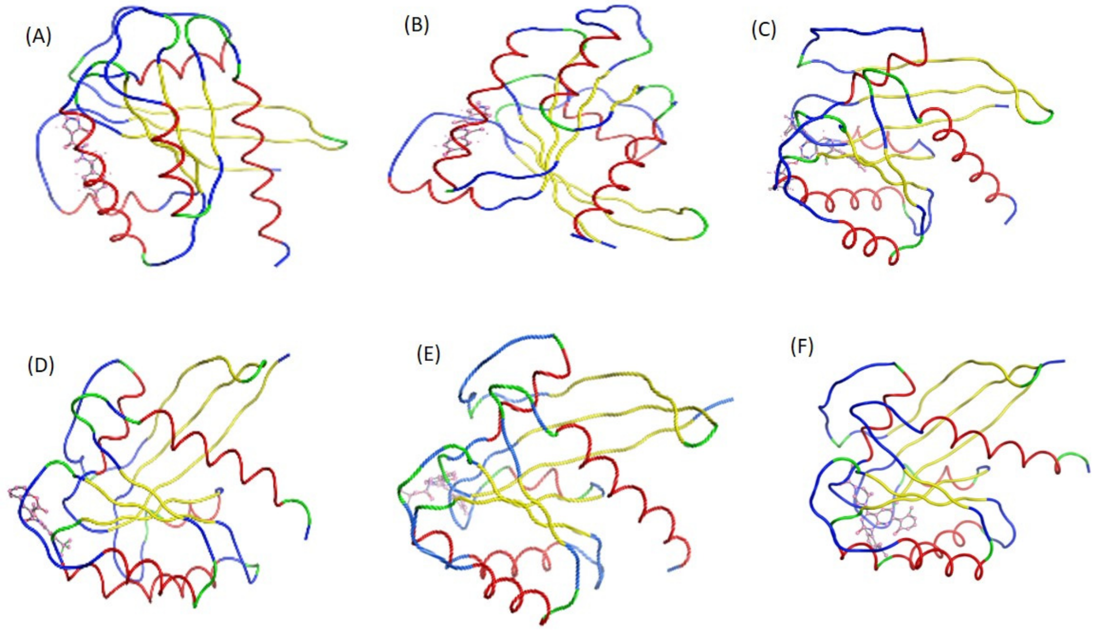

Figure S2. (A–C) indicates the complex ZINC05524764, ZINC05828661, and Control systems before MD simulation while (D–F) indicates the ZINC05524764, ZINC05828661, and Control systems after MD simulation.
